# Malate and Fumarate Extend Lifespan in *Caenorhabditis elegans*

**DOI:** 10.1371/journal.pone.0058345

**Published:** 2013-03-05

**Authors:** Clare B. Edwards, Neil Copes, Andres G. Brito, John Canfield, Patrick C. Bradshaw

**Affiliations:** Department of Cell Biology, Microbiology, and Molecular Biology, University of South Florida Tampa, Florida, United States of America; Laurentian University, Canada

## Abstract

Malate, the tricarboxylic acid (TCA) cycle metabolite, increased lifespan and thermotolerance in the nematode *C. elegans.* Malate can be synthesized from fumarate by the enzyme fumarase and further oxidized to oxaloacetate by malate dehydrogenase with the accompanying reduction of NAD. Addition of fumarate also extended lifespan, but succinate addition did not, although all three intermediates activated nuclear translocation of the cytoprotective DAF-16/FOXO transcription factor and protected from paraquat-induced oxidative stress. The glyoxylate shunt, an anabolic pathway linked to lifespan extension in *C. elegans*, reversibly converts isocitrate and acetyl-CoA to succinate, malate, and CoA. The increased longevity provided by malate addition did not occur in fumarase (*fum-1*), glyoxylate shunt (*gei-7*), succinate dehydrogenase flavoprotein (*sdha-2*), or soluble fumarate reductase F48E8.3 RNAi knockdown worms. Therefore, to increase lifespan, malate must be first converted to fumarate, then fumarate must be reduced to succinate by soluble fumarate reductase and the mitochondrial electron transport chain complex II. Reduction of fumarate to succinate is coupled with the oxidation of FADH_2_ to FAD. Lifespan extension induced by malate depended upon the longevity regulators DAF-16 and SIR-2.1. Malate supplementation did not extend the lifespan of long-lived *eat-2* mutant worms, a model of dietary restriction. Malate and fumarate addition increased oxygen consumption, but decreased ATP levels and mitochondrial membrane potential suggesting a mild uncoupling of oxidative phosphorylation. Malate also increased NADPH, NAD, and the NAD/NADH ratio. Fumarate reduction, glyoxylate shunt activity, and mild mitochondrial uncoupling likely contribute to the lifespan extension induced by malate and fumarate by increasing the amount of oxidized NAD and FAD cofactors.

## Introduction

Metabolic control of the aging process is widely accepted, yet little progress has been made in this field due to the complexity of organismal metabolism. Studies of lifespan in model organisms have yielded important roles for organelles [1], [2], especially mitochondria, in regulating the aging process. The mitochondrial electron transport chain (ETC) is the main producer of damaging reactive oxygen species in the cell and therefore has the potential to regulate lifespan as postulated by the free radical theory of aging [3]. However, recently data has accumulated that questions the theory that free radicals are the main regulators of lifespan [4], [5]. Although mitochondrial-derived oxygen radicals have been questioned as the main driving force for the aging process, changes in mitochondrial metabolism almost certainly play a role. Dietary restriction (DR), which extends lifespan [6], also delays the aging-induced ETC dysfunction in rodents [7]. DR increases the NAD/NADH ratio in many tissues [8], which stimulates mitochondrial tricarboxylic acid (TCA) cycle dehydrogenases that utilize NAD as a cofactor. The increased TCA cycle function likely necessitates increased anaplerosis, important for longevity [9].

**Figure 1:**
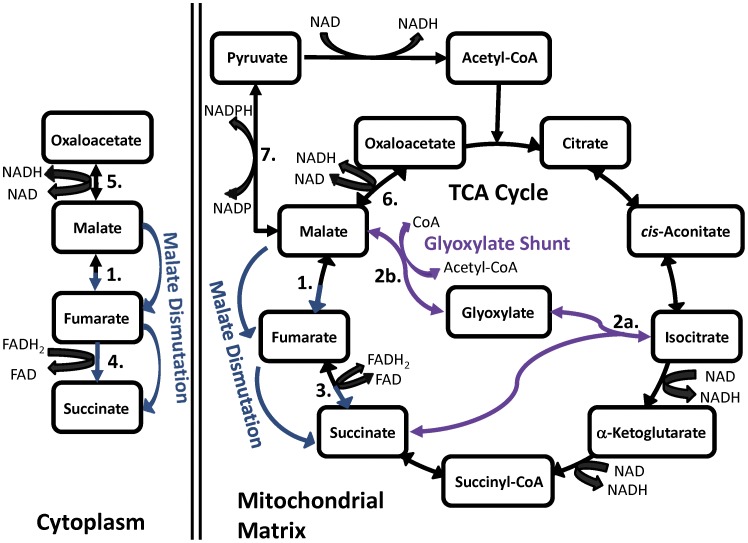
The mitochondrial TCA cycle with the glyoxylate shunt and malate dismutation. Numbered reactions are catalyzed by 1. fumarase, 2a. isocitrate lyase, 2b. malate synthase, 3. mitochondrial succinate dehydrogenase/fumarate reductase (complex II), 4. soluble fumarate reductase, 5. cytoplasmic malate dehydrogenase, 6. mitochondrial malate dehydrogenase, and 7. malic enzyme. It is unknown if the glyoxylate shunt is present in mitochondria, peroxisomes, or glyoxysomes in *C. elegans*.

Alteration of mitochondrial TCA cycle (Fig. 1) function influences lifespan in *C. elegans*. For example, RNAi knockdown of aconitase or two of the subunits of mitochondrial NAD^+^-dependent isocitrate dehydrogenase have been shown to increase lifespan [10], [11]. Mutations in the thiamine pyrophosphokinase gene, *tpk-1*, which converts thiamine to the essential co-factor thiamine pyrophosphate, essential for pyruvate and α-ketoglutarate dehydrogenases as well as several other enzymes, also extended lifespan [12]. Furthermore, addition of the TCA cycle intermediate oxaloacetate has been shown to extend lifespan in *C. elegans* through an *aak-2*/AMP kinase and *daf-16*/FOXO-dependent mechanism [13]. Supplementation with other metabolites that increase flux through the TCA cycle has also shown beneficial effects on lifespan. Addition of acetate [14] or pyruvate [15] or activation of pyruvate dehydrogenase with dichloroacetate [16] increased lifespan, while the addition of metabolites that feed more upstream into glycolysis, such as glucose or glycerol, decreased lifespan [17], perhaps due to increased methylglyoxal formation [18].

As a soil-dwelling nematode, *C. elegans* has evolved to be more metabolically flexible than many other multicellular organisms. *C. elegans* can survive anaerobically for short periods of time by utilizing a metabolic process known as malate dismutation (Fig. 1) or the phosphoenolpyruvate carboxykinase (PEPCK)-succinate pathway [19], [20]. Here, a portion of the intracellular malate is converted to fumarate and then to succinate, which can be excreted from the cell. This process leads to the oxidation of reducing equivalents providing NAD and FAD essential for cellular metabolism. *C. elegans* also has a glyoxylate shunt, not present in mammals, that converts isocitrate and acetyl-CoA to succinate, malate, and CoA using glyoxylate as an intermediate (Fig. 1) [21]. This shunt bypasses two NADH and CO_2_ generating steps in the TCA cycle, conserving NAD levels and preventing carbon loss, which is advantageous for biosynthetic reactions in the cell. The glyoxylate shunt is upregulated in many long-lived *C. elegans* mutants [22].

In this report, we tested the effect of added succinate, fumarate, and malate on *C. elegans* lifespan, and determined the effects on mitochondrial function, redox status, and determined which metabolic enzymes and longevity pathways were necessary for lifespan extension.

## Methods

### *C. elegans* Culture

The N2 strain of *C. elegans* at a concentration of 2,000 worms per mL were grown in aerated liquid S medium containing 2 g of HT115(DE3) bacteria per 100 mL of media and 10 µL of antifoam 204 (Sigma) per 100 mL of media. The cultures were maintained in either a 250 mL volume in 375 mL clear longneck 6 cm-wide glass bottles or in a 100 mL volume in 100 mL round glass media storage bottles placed in a thermoelectric cooler/warmer (www.kotulas.com) at 20°C. Cultures were aerated with one line of a 20–60 gallon double outlet aquarium air pump (Aqua Culture) connected with tubing containing a check valve to prevent backflow. The bottles were sealed with Parafilm and a hole was drilled in the lid of the 100 mL bottles for the aeration tubing. Deionized water or S medium was added back every three days to compensate for evaporation, and the media and the bacteria were replaced every 6 days.

### Chemicals

L-malic acid was purchased from Chem-Impex International. Succinic acid and fumaric acid were obtained from Fisher Scientific. 5-fluoro-2′-deoxyuridine (FUdR) was obtained from Research Products International Corp. Sodium hydroxide was added to metabolite stock solutions to obtain a pH of 7.0.

### Lifespan Measurements

The worms were bleach-synchronized as follows: 2 mL of 6% NaOCl were mixed with 1 mL of 5 M NaOH per 7.5 mL of concentrated worm suspension, and shaken for 4–7 minutes until the carcasses dissolved as monitored by direct observation. The remaining eggs were then washed 3 times by pelleting at ∼1150 *g* for 2 minutes at room temperature, followed by aspiration of the supernatant and resuspension in 50 mL of 0.1 M NaCl. A final pellet of eggs was obtained by centrifugation at ∼1150 *g* for 2 minutes at room temperature, followed by aspiration of the supernatant. Eggs were then added to a 250 mL liquid culture, as described above. For experiments without RNAi treatment, bacteria were heat killed at 80°C for 60 minutes. The worms were cultured at 20°C and monitored until they reached adulthood (∼72 h), at which time FUdR was added to a final concentration of 400 uM. Worm viability was scored every two days by taking ten 10 µL drops (initially ∼20 worms per drop) of the culture and counting the living worms under a microscope. The average number of living worms was then calculated. S medium or deionized water containing 10 mM malate, succinate, or fumarate was added back every three days to compensate for metabolism of the compounds and evaporation, and S medium containing FUdR and bacteria was replaced every 6 days. At least two replicates of each experiment were performed.

AMPK kinase *aak-2* mutant worms grew slowly in liquid media, so this strain was maintained on NGM agar plates [23] and lifespan experiments were performed on NGM agar plates with 400 uM FUdR under standard conditions [24]. Several of the lifespan experiments (*sdha-2* RNAi, F48E8.3 RNAi, W06B3.1 RNAi, *qns-1* RNAi, *fum-1* RNAi+fumarate, *gei-7* RNAi+fumarate, α-ketoglutarate, aspartate, glyoxylate, *hsf-1(sy441),* and *hif-1(ia4)* were conducted in liquid media using 0.4 µM or 3 µM transparent cell culture inserts (BD Falcon #353180 and #353181) in 12-well and 24-well microplates as first described in [25] on an orbital shaker at 135 rotations/min at 20°C. 12-well microplates were found preferable due to the easier visualization of the worms through the suspension of bacteria after swirling the microplate. 3 µM inserts were found preferable due to the increased *E. coli* permeability. Initially 1.25 mL of S-medium containing 9×10^9^ HT115(DE3) *E. coli* per mL was placed in each well of a 12-well microplate. Then bleach synchronized worm eggs were suspended at a concentration of 100–200 eggs/mL in S-medium containing 9×10^9^ HT115(DE3) *E. coli* per mL. Lastly, a cell culture insert was placed in each well followed by 0.25 mL of the egg/bacterial suspension (25–50 eggs) into each insert (*n* = 4–6 wells per condition).

### Protein Assay

Protein was assayed essentially as in [26]. Briefly, one mL of worms suspended in S medium or M9 medium was snap frozen in liquid nitrogen and stored at −10°C until analysis. For analysis 500 µL of a worm suspension was sonicated on ice, using a W-380 sonicator (Heat Systems-Ultrasonics, Inc.) (5-second pulses, 50% duty cycle, max power, 12 pulses). 1.5 mL of 1∶1 ethanol/acetone was added and the suspension was vortexed, and incubated for 30 minutes at 4°C. The tube was then centrifuged at 15,000×*g* for 10 minutes at room temperature. The supernatant was decanted, and the tube was inverted on a paper towel while the pellet dried. The pellet was then resuspended in 180 µL of 1 N NaOH, and incubated at 70°C for 25 minutes to degrade lipids that could have interfered with analysis. The NaOH was then diluted with 1.26 mL of deionized water and 360 µL of 10% SDS. The sample was then mixed by inversion and centrifuged at 1,500×*g* for 2 minutes at room temperature. The protein content of the supernatant was then analyzed by the BCA assay (Pierce) according to the manufacturer’s protocol.

### NAD, NADH, NAD, and NADPH Measurements

The *C. elegans* MH1317 strain having genotype kuIs29 [unc-119(+) egl-13::GFP(pWH17)] V was used. Worms were synchronized and cultured with heat-killed OP50 *E. coli* as food in the presence of no treatment, 10 mM malate or 10 mM succinate. On day 4 of the lifespan a 2 mL aliquot of each culture was snap frozen in liquid nitrogen. The samples were thawed and 50 µL was added in duplicate to the wells of a 96-well plate. NAD, NADH, NAD, and NADPH measurements were performed using the Elite Fluorimetric NAD/NADH Ratio Assay and Elite NADP/NADPH Ratio Assay Kits (eENZYME, LLC), according to the manufacturer’s instructions. Fluorescence was normalized by the GFP expression of each sample.

### Thermotolerance Assay

A synchronized population of N2 *C. elegans* eggs was obtained as described above for the lifespan measurements. Eggs were placed in an aerated longneck glass bottle filled to 250 mL with liquid S medium and 4 g of HT115(DE3) *E. coli* with and without 10 mM of malate, fumarate, or succinate. On day 5 of the lifespan, the worms were removed and diluted to approximately 10 worms per well in a 96 well microplate (control *n* = 219, malate *n* = 213). The 96 well microplate containing malate-treated and untreated worms was placed in an incubator at 38°C. Worms were scored for movement as a marker of survival every 20–30 minutes for 430 minutes.

### GST-4::GFP Fluorescence Analysis

*C. elegans* of strain CL2166 having genotype dvIs19[pAF15(gst-4::GFP::NLS)] as described in [27] were used. Approximately 300 age-synchronized worms were grown in cell culture inserts in liquid culture medium containing HT115(DE3) *E. coli* as food as described above. Cultures were supplemented with 10 mM succinate, 10 mM malate, or 10 mM fumarate on day 1 of the lifespan. 10 mM paraquat was added on day 4. 24 hours later on day 5 approximately 20 adult worms from each treatment group were removed and assayed by fluorescence microscopy. Worms in the images were analyzed for fluorescence intensity following background subtraction using NIH ImageJ software version 1.44p.

### DAF-16::GFP and SKN-1::GFP Nuclear Translocation Experiments

*C. elegans* strains N2, TJ356 (DAF-16::GFP), and LD1008 (SKN-1::GFP) were bleach synchronized and eggs were placed in 3 µM cell culture inserts with heat-killed HT115(DE3) *E. coli* in shaken 12-well plates untreated or treated with 10 mM malate, 10 mM fumarate, or 10 mM succinate (3 wells per treatment). On day 4 of the lifespan, worms were chilled on ice to slow movement and 40–50 worms per treatment group were photographed and analyzed for nuclear translocation.

### Thrashing and Pharyngeal Pumping Measurements

For the thrashing assays N2 worms were grown on NGM agar with live HT115(DE3) control *E. coli* or HT115(DE3) *E. coli* expressing RNAi to malic enzyme (*men-1*) essentially as in [28]. Some worms were grown in the presence of heat-killed HT115(DE3) *E. coli* with 10 mM malate, 10 mM fumarate, or 10 mM succinate, or no addition. Worms were transferred to 50 µL of S medium. After a one minute recovery period thrashes, defined as changes in the direction of bending at the mid body, were counted for 30 seconds (*n* = 8 for the control N2 worms and the N2 worms feeding on malic enzyme (*men-1*) RNAi-expressing bacteria and *n* = 16 for malate, fumarate, succinate, and control treated N2 worms). Pharyngeal pumping assays were performed essentially as in [29]. Briefly, age-synchronized eggs from N2 worms were placed on 6 cm agar plates seeded with OP50 *E. coli* suspended in S medium with or without 10 mM malate. Video of 3 day old worms (*n* = 16 for each group) was recorded with a Scopetek 3.2 megapixel microscope eyepiece camera at a resolution of 1028×764 pixels and quality setting of 50 out of 100 in black and white. Full pumps were manually counted for 20 seconds during reduced speed video playback using the VLC media player.

### Oxygen Consumption Measurements

N2 worms were grown using heat killed HT115(DE3) *E. coli* as food for 4 days with and without 10 mM malate and separated from the bacteria by filtering nine times through a 10 micron polypropylene or nylon mesh (www.amazonsupply.com) attached to a 30 mL syringe. Worms were washed off the mesh and resuspended in M9 medium. The average concentration of worms was obtained by taking ten 10 µL drops and counting the number of living worms in each drop. The volume of the culture was then adjusted to obtain a final concentration of 2 worms per µL. 350 µL of the worm suspension was then added to the chamber of a Clark oxygen electrode (MT200A chamber, Strathkelvin Instruments) and the respiration was monitored for approximately 3 minutes. The respiratory rate was normalized to protein content by performing a protein assay on the worm suspension.

### ATP Assays

One mL samples of the N2 *C. elegans* cultures grown with either heat-killed *E. coli* or live RNAi-expressing *E. coli* were snap frozen on day 4 of the lifespan in liquid nitrogen. The samples were thawed and then 50 µL was added to a well of a 96-well microplate in a 1∶1 ratio with 50 µL of CellTiter Glo solution (Promega, Madison, WI). The plate was shaken for 2 minutes and then incubated at room temperature for 10 minutes. Luminescence of the samples was measured in a Biotek Synergy 2 microplate reader. ATP levels were obtained through the use of a standard curve.

### Mitochondrial Membrane Potential Determination

N2 *C. elegans* were bleach synchronized and 500 eggs were placed in each well of a 12-well shaken microplate along with heat-killed HT115(DE3) *E. coli*. 24 hours later each well was treated with 100 nM tetramethylrhodamine ethylester (TMRE). In addition specific wells were treated with 10 mM malate, 10 mM fumarate, 10 mM succinate, or 10 µM FCCP (trifluorocarbonylcyanide phenylhydrazone) (3 wells per treatment). 24 hours following treatment the worms for each treatment group were washed with 10 mL of S-medium and resuspended in 5 mL of S-medium. 100 µL of worms were added to each well of a 96-well microplate (*n* = 6) and fluorescence was measured using a 540/30 nm excitation filter and a 590/35 nm emission filter.

### Statistical Analysis

Kaplan-Meier survival analysis and log-rank tests were performed using Sigmaplot version 11.0. Student’s t-tests were used in other analyses.

## Results

### Malate Extends Lifespan in WT but not *eat-2*, *daf-16, sir-2.1, or hsf-1* Mutant Worms

**Figure 2:**
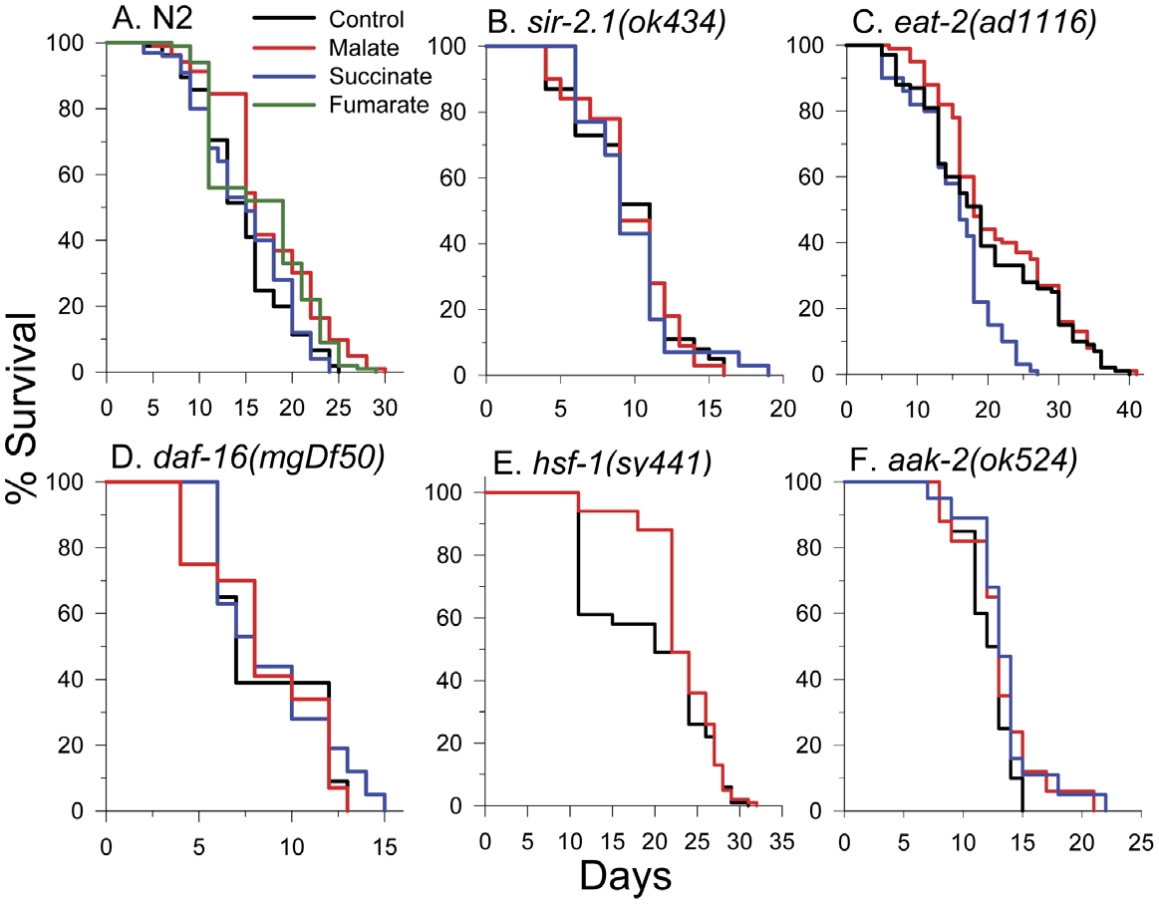
Lifespan analysis of *C. elegans* strains in the presence of TCA cycle intermediates. 10 mM concentrations of malate, succinate, or fumarate were present where indicated. (**A**) Malate (log-rank *p*<0.001) or fumarate (log-rank *p*<0.001), but not succinate (log-rank *p* = 0.72) extended lifespan of N2 worms. (**B**) Neither malate (log-rank *p* = 0.97) nor succinate (log-rank *p* = 0.88) extended lifespan of *sir-2.1(ok434)* worms. (**C**) No effect of malate treatment (log-rank *p* = 0.22) on lifespan and decreased lifespan with succinate treatment (log-rank *p*<0.001) in *eat-2(ad1116)* worms. (**D**) Neither malate (log-rank *p* = 0.83) nor succinate (log-rank *p = *0.22) extended lifespan of *daf-16(mgDf50)* worms. (**E**) 10 mM malate (log-rank *p* = 0.08) did not extend the lifespan of HSF-1 mutant *hsf-1(sy441)* worms. (**F**) Neither 10 mM malate (log-rank *p* = 0.18) nor 10 mM succinate (log-rank *p* = 0.10) extended lifespan of AMPK mutant *aak-2(ok524)* worms.

In Fig. 2A we show that the addition of 10 mM L-malate and 10 mM fumarate, but not 10 mM succinate, to the growth medium of *C. elegans* increased lifespan. A summary of all lifespan experiments is shown in Table 1. Malate increased mean lifespan by 14% and the increase was consistently observed (p<0.001) in nine total replicates using either live *E. coli* (*n* = 6) or heat killed *E. coli* (*n* = 3) as the food source. The increased lifespan was not due to reduced food intake from diminished pharyngeal pumping as malate treated (164 pumps per minute ±4 SEM) showed similar rates as control (168±3 SEM) worms (*p* = 0.37). Fumarate increased mean lifespan by 16% (p<0.001 *n* = 4). Interestingly, a mass spectrometry-based metabolomics analysis identified 188 total worm metabolites and indicated that there was a 2-fold reduction in fumarate levels with aging in *C. elegans* (data not shown). Therefore, added fumarate or malate may compensate for altered TCA cycle function in aged worms. Like succinate, the TCA cycle intermediate α-ketoglutarate failed to extend lifespan (Fig. S1). Aspartate (Fig. S2), a metabolite of the mitochondrial malate-aspartate NADH shuttle also failed to induce lifespan extension.

**Table 1:** Summary of lifespan experiments.

Strain			Treatment		% of untreated mean lifespan		% of N2 mean lifespan	Worms counted*^a^*		Log-rankp-value	
N2			malate		114		114		1,425	<0.001	
N2			fumarate		116		116		469	<0.001	
N2			succinate		96		96		163	0.72	
N2*^c^*			α-ketoglu*^b^*		97		97		65	0.21	
N2*^c^*			aspartate		102		102		188	0.57	
N2*^c^*			glyoxylate		96		96		45	0.35	
N2 (agar)			malate		110				51	0.04	
*sir-2.1(ok434)*							65		45	<0.001	
*sir-2.1(ok434)*			malate		102				36	0.97	
*sir-2.1(ok434)*			succinate		102				25	0.88	
*eat-2(ad1116)*							120		212	<0.001	
*eat-2(ad1116)*			malate		110				348	0.22	
*eat-2(ad1116)*			succinate		82				193	<0.001	
*daf-16(mgDf50)*							59		54	<0.001	
*daf-16(mgDf50)*			malate		97				56	0.83	
*daf-16(mgDf50)*			succinate		103				43	0.22	
*aak-2(ok524)* (agar)							74		20	<0.001	
*aak-2(ok524)* (agar)			malate		108				17	0.18	
*aak-2(ok524)* (agar)			succinate		111				19	0.10	
*hsf-1(sy441)^c^*							91		181	0.46	
*hsf-1(sy441)^c^*			malate		121				191	0.08	
*hif-1(ia4)^c^*							72		242	<0.001	
*hif-1(ia4)^c^*			malate		125				299	<0.001	
*hif-1(ia4)^c^*			fumarate		110				313	0.02	
N2	*skn-1*	RNAi					70		126	<0.001	
N2	*skn-1*	RNAi		malate		109			95	0.03	
N2	*men-1*	RNAi					53		224	<0.001	
N2	*men-1*	RNAi	malate		130				346	<0.001	
N2	*mdh-1*	RNAi					66		642	<0.001	
N2	*mdh-1*	RNAI	malate		126				800	<0.001	
N2	F46E10.10	RNAi					85		230	<0.001	
N2	F46E10.10	RNAi	malate		108				180	0.006	
N2	*fum-1*	RNAi					66		120	<0.001	
N2	*fum-1*	RNAi	malate		101				125	0.49	
N2	*fum-1^c^*	RNAi	fumarate		118				545	<0.001	
N2	*gei-7*	RNAi					62		183	0.02	
N2	*gei-7*	RNAi	malate		112				188	0.24	
N2	*gei-7^c^*	RNAi	fumarate		91				115	0.02	
N2	*flad-1*	RNAi					71		280	<0.001	
N2	*flad-1*	RNAi	malate		160				280	<0.001	
N2	*sdha-1*	RNAi					78		466	<0.001	
N2	*sdha-1*	RNAi	malate		120				267	<0.001	
N2	*sdha-2^c^*	RNAi					92		393	0.12	
N2	*sdha-2^c^*	RNAi	malate		95				290	0.95	
N2	F48E8.3*^c^*	RNAi					79		300	<0.001	
N2	F48E8.3*^c^*	RNAi	malate		90				270	0.002	
N2	*qns-1^c^*	RNAi					76		177	<0.001	
N2	*qns-1^c^*	RNAi	malate		113				185	0.07	
N2	W06B3.1*^c^*	RNAi					94		188	0.46	
N2	W06B3.1*^c^*	RNAi	malate		91				163	0.07	

Malate addition was unable to extend the lifespan of *sir-2.1(ok434)* mutant worms (Fig. 2B). SIR-2.1 is a sirtuin family member and is the closest worm homolog of the mammalian SirT1 NAD-dependent protein deacetylase [30]. In Fig. 2C, it is shown that malate did not extend the lifespan of long-lived *eat-2(ad1116)* worms that have reduced pharyngeal pumping rates and are a model of dietary restriction. Treatment with succinate blocked the lifespan extending effects of dietary restriction (DR) in *eat-2* worms, as the maximal lifespan was similar as the N2 control strain and much shorter than untreated or malate treated *eat-2* worms. Fig. 2D shows that malate did not extend the lifespan of *daf-16(mgDf50)* worms. Therefore, DAF-16 is required for malate-induced lifespan extension. DAF-16 is the worm homolog of mammalian FOXO transcription factors and is required for lifespan extension in several mutant strains, most notably in reduced insulin receptor signaling *daf-2* mutant worms. Malate also failed to significantly extend the lifespan of heat shock factor-1 mutant, *hsf-1(sy441)* worms (log-rank *p* = 0.08) (Figure 2E), although a protective effect occurred early in life. HSF-1 is required for lifespan extension that occurs in *daf-2* mutants [31] and in some dietary restriction regiments [32], [33].

### Malate Increased Lifespan Robustly in *hif-1* Mutant Worms and Slightly in *aak-2* and *skn-1* RNAi Worms

**Figure 3:**
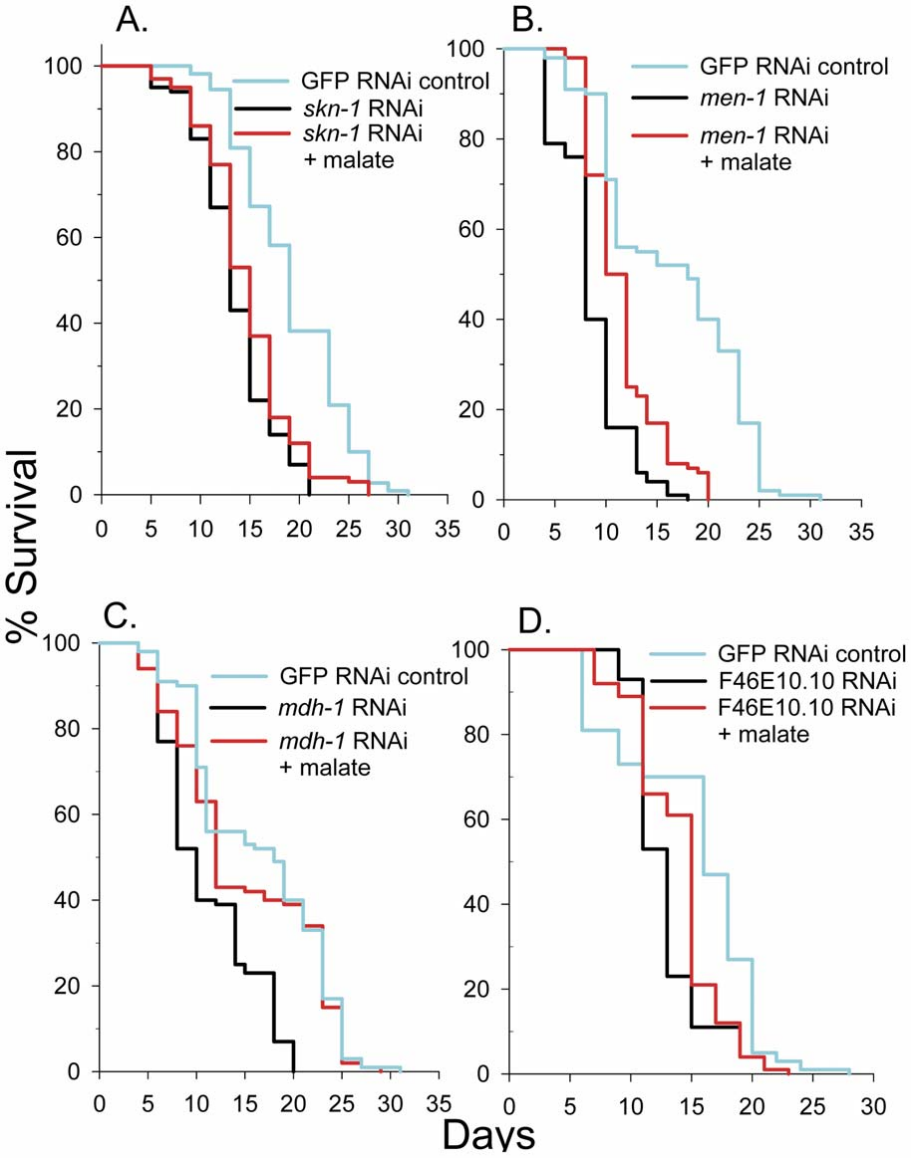
Adding malate to the media increased lifespan of *C. elegans* RNAi knockdown strains. (**A**) 10 mM malate increased lifespan of *skn-1* RNAi knockdown N2 worms (log-rank *p* = 0.03). (**B**) 10 mM malate increased the lifespan of malic enzyme (*men-1*) RNAi knockdown N2 worms (log-rank *p*<0.001). (**C**) 10 mM malate increased the lifespan of mitochondrial malate dehydrogenase (*mdh-1*) RNAi knockdown N2 worms (log-rank *p*<0.001). (**D**) 10 mM malate increased the lifespan of cytoplasmic malate dehydrogenase F46E10.10 RNAi knockdown N2 worms (log-rank *p* = 0.006).

Malate and fumarate treatments resulted in increases in the lifespan of hypoxia inducible factor-1 mutant, *hif-1(ia4)* worms (Fig. S3). HIF-1 functions downstream of Tor kinase and is necessary for the lifespan extension that occurs in mitochondrial mutants [34], [35]. Malate and succinate treatments did not significantly increase the mean lifespan of *aak-2(ok524)* AMP kinase (AMPK) mutant worms (Fig. 2F). But caution should be used when making conclusions from this data, due to the low number of worms used in these experiments. AMPK has been shown to be necessary for lifespan extension in worms following oxaloacetate treatment [13], resveratrol treatment, and under certain DR conditions [32]. SKN-1 is the *C. elegans* homolog of mammalian Nrf transcription factors involved in cellular detoxification, stress defense, and longevity. Malate extended the lifespan of *skn-1* RNAi nematodes by 9% (Fig. 3A). Consistent with this, malate, fumarate, and succinate all failed to induce nuclear localization of SKN-1::GFP (data not shown).

### Malic Enzyme or Malate Dehydrogenase Knockdown did not Block Malate-Induced Lifespan Extension

Malic enzyme catalyzes the conversion of malate to pyruvate and carbon dioxide with the concurrent reduction of NADP to NADPH. To determine if this reaction is essential for the lifespan extension elicited by malate, we knocked down the sole malic enzyme gene in *C. elegans, men-1* by RNAi and determined the effects of malate addition on lifespan. As shown in Fig. 3B, the mean lifespan of *men-1* RNAi knockdown worms was only 53% of controls, but malate addition still extended mean lifespan by 30%, suggesting that high malic enzyme activity is not important for malate-induced lifespan extension. Interestingly, malic enzyme RNAi knockdown caused an increased rate of body wall muscle contractility (210 body bends per minute ±12 SEM) when compared to control (147 body bends per minute ±7 SEM) as measured by a thrashing assay (*p*<0.001).

Malate dehydrogenase catalyzes the reversible conversion of malate to oxaloacetate with the concurrent reduction of NAD to NADH. There are two confirmed malate dehydrogenase genes in *C. elegans*. The *mdh-1* gene codes for a mitochondrial isoform, while the other gene, F46E10.10, encodes a cytoplasmic isoform. F46E10.10 is upregulated in long-lived dauer and *daf-2* worms [19] as well as long-lived mitochondrial mutants [36]. Oxaloacetate, the product of the malate dehydrogenase reaction has been shown to extend lifespan in *C. elegans*. We first knocked down the mitochondrial malate dehydrogenase, *mdh-1,* and determined the effects on lifespan in the absence and presence of malate (Fig. 3C). The mean lifespan of *mdh-1* knockdown worms was 66% of controls, but malate addition extended mean lifespan of the RNAi treatment by 26%, nearly back to that observed in the control worms. We then knocked down F46E10.10 and performed lifespan analysis with and without malate (Fig. 3D). Knockdown worms showed a mean lifespan 85% of controls and malate addition extended mean lifespan of the F46E10.10 knockdown worms by 8%.

### The Glyoxylate Shunt is Required for Lifespan Extension Induced by Malate or Fumarate

**Figure 4:**
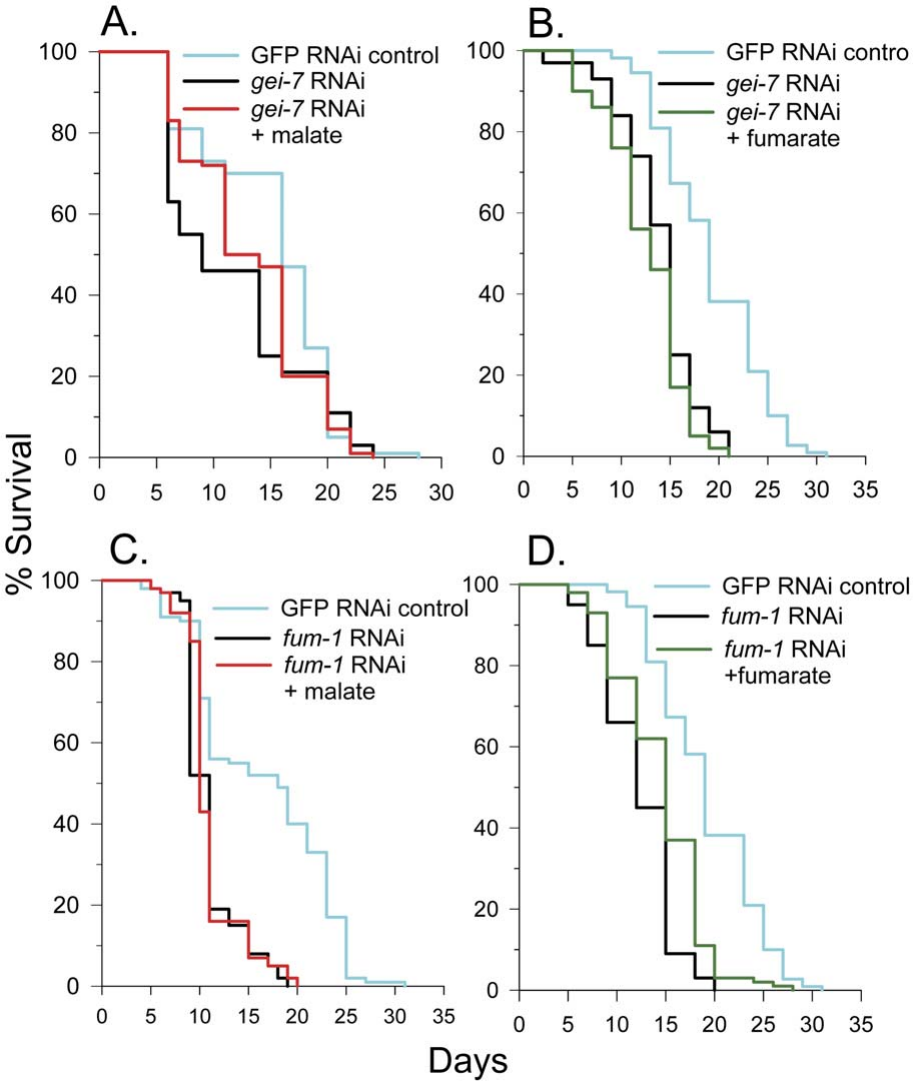
Malate-induced lifespan extension requires fumarase and the glyoxylate cycle. (**A**) 10 mM malate treatment did not alter the lifespan of glyoxylate cycle *gei-7* RNAi knockdown N2 worms (log-rank *p* = 0.24). (**B**) 10 mM fumarate treatment decreased the lifespan of glyoxylate cycle *gei-7* RNAi knockdown N2 worms (log-rank *p* = 0.02). (**C**) 10 mM malate treatment did not alter the lifespan of fumarase (*fum-1*) RNAi knockdown N2 worms (log-rank *p* = 0.49). (**D**) 10 mM fumarate treatment increased the lifespan of fumarase (*fum-1*) RNAi knockdown N2 worms (log-rank *p*<0.001).

The glyoxylate shunt is composed of two enzymes, isocitrate lyase and malate synthase. In *C. elegans* these enzymes are fused into one bifunctional protein named GEI-7 or ICL-1. Isocitrate lyase reversibly converts isocitrate into succinate and glyoxylate. Malate synthase catalyzes the reversible synthesis of malate and CoA from glyoxylate and acetyl-CoA (Fig. 1). Since malate can be metabolized by the glyoxylate shunt we determined the lifespan of *gei-7* RNAi worms in the absence and presence of malate. As shown in Fig. 4A, the mean lifespan of *gei-7* RNAi knockdown worms was 62% of controls and the lifespan was not statistically different in the presence of malate (log-rank *p* = 0.24). Fumarate addition also did not increase lifespan and even decreased the lifespan of the *gei-7* RNAi knockdown worms by 9% (Fig. 4B). Therefore, the glyoxylate shunt appears to be essential for the lifespan extension mediated by malate or fumarate treatment. Interestingly, 10 mM glyoxylate failed to extend lifespan (Fig. S4), suggesting a limited conversion of glyoxylate to malate in the worms under these culture conditions or alternatively, that the acetyl-CoA consumed during conversion of glyoxylate to malate may prevent lifespan extension.

### Fumarase is Required for Lifespan Extension Induced by Malate but not by Fumarate

Fumarase catalyzes the reversible conversion of fumarate to malate and is dually targeted to both the cytoplasm and mitochondria [37]. We performed a lifespan assay using fumarase (*fum-1*) RNAi knockdown worms in the presence and absence of malate (Fig. 4C). The mean lifespan of *fum-1* knockdown worms was 66% of controls. Strikingly, *fum-1* knockdown prevented malate from increasing lifespan (log-rank, *p* = 0.49). But in contrast to malate addition, fumarate addition, which is still able to be converted to succinate, did extend lifespan by 18% in *fum-1* RNAi knockdown worms (Fig. 4D). Therefore, added malate must be metabolized by fumarase to form fumarate, running a portion of the TCA cycle backwards, to extend lifespan.

### Malate Increased Lifespan in *sdha-1* RNAi Knockdown Worms, but not in *sdha-2* and F48E8.3 RNAi Knockdown Worms

There are three separate fumarate reductase isoforms in *C. elegans*. Two of them, *sdha-1* and *sdha-2*, are exchangeable subunits of the mitochondrial fumarate reductase/succinate dehydrogenase complex II of the respiratory chain and the other is a soluble cytoplasmic fumarate reductase F48E8.3. There has been a report of a large decrease in *sdha-2* expression in long-lived dauer larvae [20], but another group found no change [19]. SDHA-2 protein levels were downregulated in long-lived *eat-2* worms [38]. *Sdha-1* expression levels were unchanged [20] or slightly decreased [19] in dauers, while F48E8.3 was strongly upregulated [19], [20]. F48E8.3 protein levels were not changed in *eat-2* worms [38]. Proteomics analysis showed an increase in *sdha-1* levels with aging (data not shown). It was hypothesized that the *sdha-1*/*sdha-2* ratio may influence lifespan by regulating the relative fumarate reductase to succinate dehydrogenase activity of complex II [19]. However, others hypothesized that complex II flavoprotein phosphorylation may play a role in controlling the relative fumarate reductase to succinate dehydrogenase activities [39].

**Figure 5:**
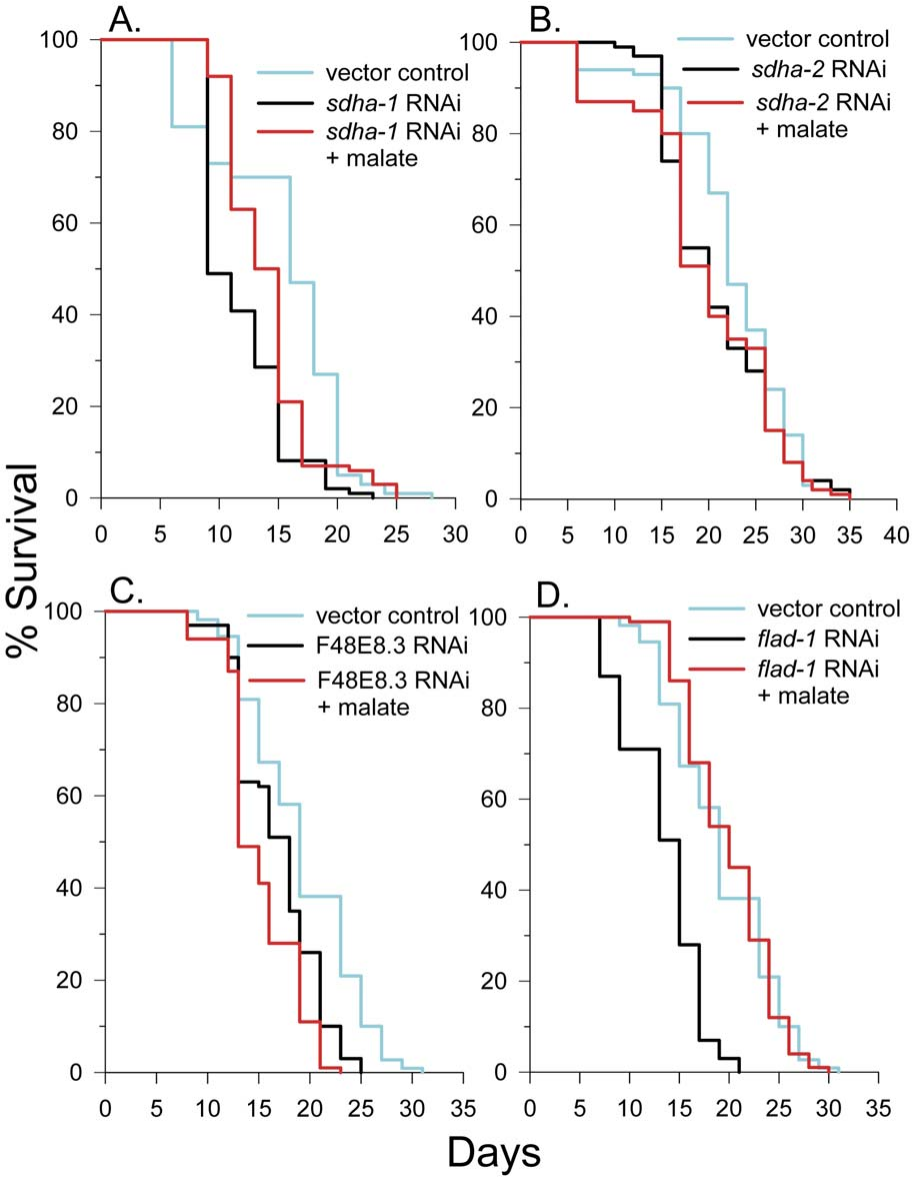
Malate treatment did not increase the lifespan of *sdha-2* and F48E8.3 RNAi knockdown worms. (**A**) 10 mM malate increased the lifespan of complex II flavoprotein (*sdha-1*) RNAi knockdown N2 worms (log-rank *p*<0.001). (**B**) 10 mM malate did not increase the lifespan of complex II flavoprotein (*sdha-2*) RNAi knockdown N2 worms (log-rank *p* = 0.95). (**C**) 10 mM malate decreased the lifespan of soluble fumarate reductase F48E8.3 RNAi knockdown N2 worms (log-rank *p* = 0.002). (**D**) 10 mM malate increased the lifespan of FAD synthase (*flad-1*) RNAi knockdown N2 worms (log-rank *p*<0.001).

To determine if *sdha-1* plays a role in lifespan extension mediated by malate, we knocked it down by RNAi treatment and determined the lifespan in the absence and presence of malate. As shown in Fig. 5A, *sdha-1* knockdown worms had a mean lifespan 78% of controls and malate treatment increased mean lifespan by 20%. The data indicate that *sdha-1* does not likely play a role in lifespan extension by malate. We next knocked down *sdha-2* by RNAi and determined the effect of malate treatment. Malate addition did not significantly alter the lifespan (log-rank *p* = 0.95) (Fig. 5B). We also knocked down the soluble fumarate reductase F48E8.3 by RNAi. These worms only lived 79% as long as controls. Once again, malate addition did not extend lifespan and even caused a 10% decrease in lifespan. (Fig. 5C). These experiments further confirm the role of fumarate reduction and the malate dismutation pathway in the lifespan extending effects of malate.

### The Effect of Malate on Lifespan in Flavin Adenine Dinucleotide (FAD) Synthase RNAi Knockdown Worms

One possible difference between succinate and malate metabolism and their different effects on lifespan is that following malate conversion to fumarate, fumarate is metabolized to succinate by fumarate reductase to increase the FAD/FADH_2_ ratio in the cell, while succinate conversion to fumarate has the opposite effect on the ratio. To gain insight into a possible role for FAD levels on lifespan extension by malate, we determined if malate could extend lifespan when the *flad-1* gene encoding FAD synthase, the terminal step in FAD synthesis, was knocked down by RNAi. As shown in Fig. 5D, *flad-1* RNAi knockdown had a mean lifespan of 71% of the control, and malate treatment completely restored the lifespan back to that of the control. Therefore, FAD levels do not appear to be the limiting factor for malate-mediated lifespan extension. It is possible, however, that a low FAD/FADH_2_ ratio limits normal *C. elegans* lifespan under these growth conditions and malate treatment increases this ratio to increase lifespan.

### Malate, Fumarate, and Succinate Treatment Increased Stress Resistance

**Figure 6:**
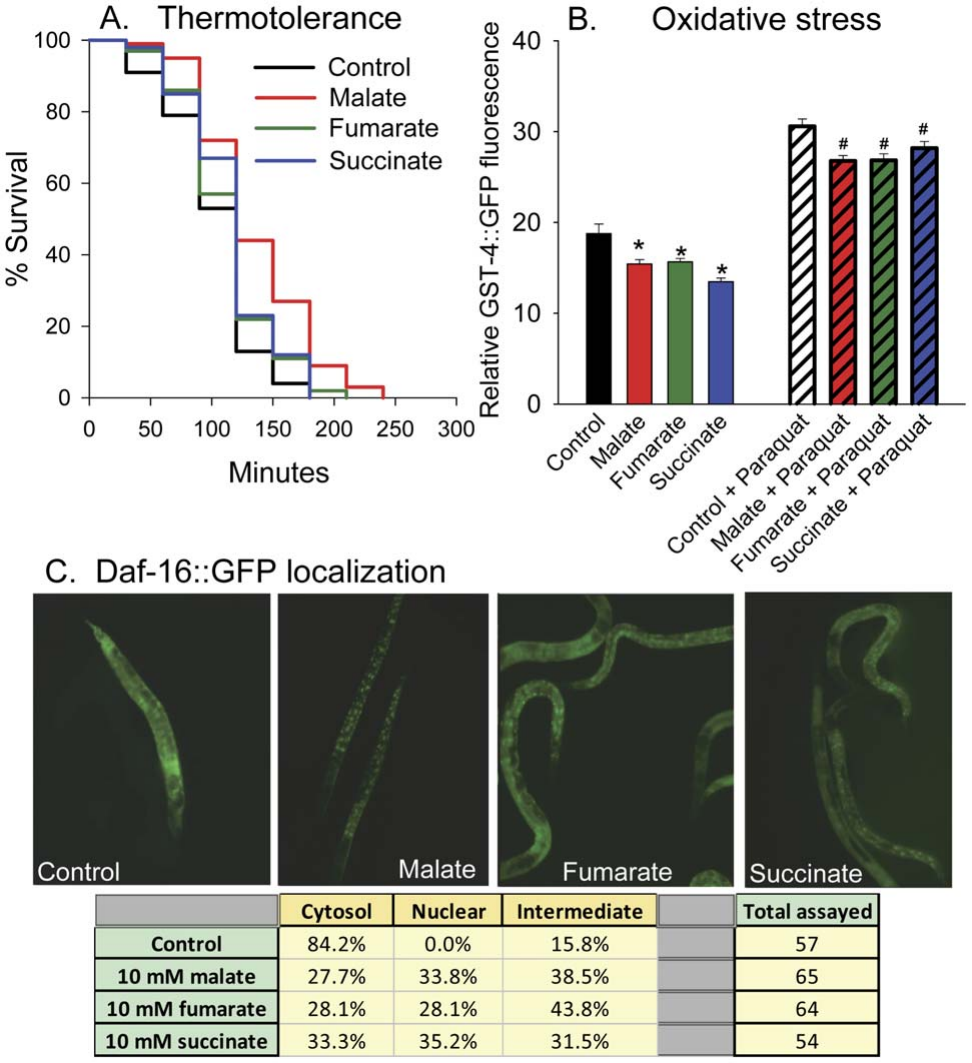
Effects of malate, fumarate, and succinate on thermotolerance, oxidative stress, and DAF-16::GFP nuclear translocation. (**A**) 10 mM malate increased thermotolerance (log-rank *p*<0.001), while 10 mM succinate (log-rank *p* = 0.03) and 10 mM fumarate (log-rank *p* = 0.12) had smaller protective effects. *C. elegans* were grown at 20°C and then upshifted to 38°C. (**B**) 10 mM malate, fumarate, or succinate treatment decreased GST-4::GFP fluorescence in the absence (*p<0.05 compared to untreated N2) and in the presence of 10 mM paraquat (# *p*<0.05 compared to paraquat treated N2). (**C**) 10 mM malate, fumarate, or succinate treatment increased the nuclear translocation of DAF-16::GFP.

Since many treatments and mutations that extend lifespan also increase stress resistance, we determined if malate, fumarate, or succinate could also increase the thermotolerance of the worms or decrease oxidative stress. As shown in Fig. 6A, malate increased the thermotolerance, the survival time of the worms at 38°C, by 32%, while succinate (log-rank *p* = 0.03) and fumarate (log-rank *p* = 0.12) were less protective, only increasing thermotolerance by 13% and 10% respectively. The transcription factor SKN-1/Nrf is upregulated in *C. elegans* in response to oxidative stress and activates transcription of antioxidant genes such as glutathione-S-transferase-4 (*gst-4*). By monitoring the fluorescence of a *gst-4::gfp* oxidative stress reporter worm strain [27], we found that malate, fumarate, and succinate all decreased endogenous oxidative stress and the oxidative stress following treatment with paraquat, a stimulator of mitochondrial reactive oxygen species production (Fig. 6B). Consistent with this protection, malate, fumarate, and succinate treatment all induced the nuclear translocation of DAF-16::GFP (Fig. 6C).

### Malate Treatment Increased the NAD/NADH Ratio and Decreased the NADP/NADPH Ratio

**Figure 7:**
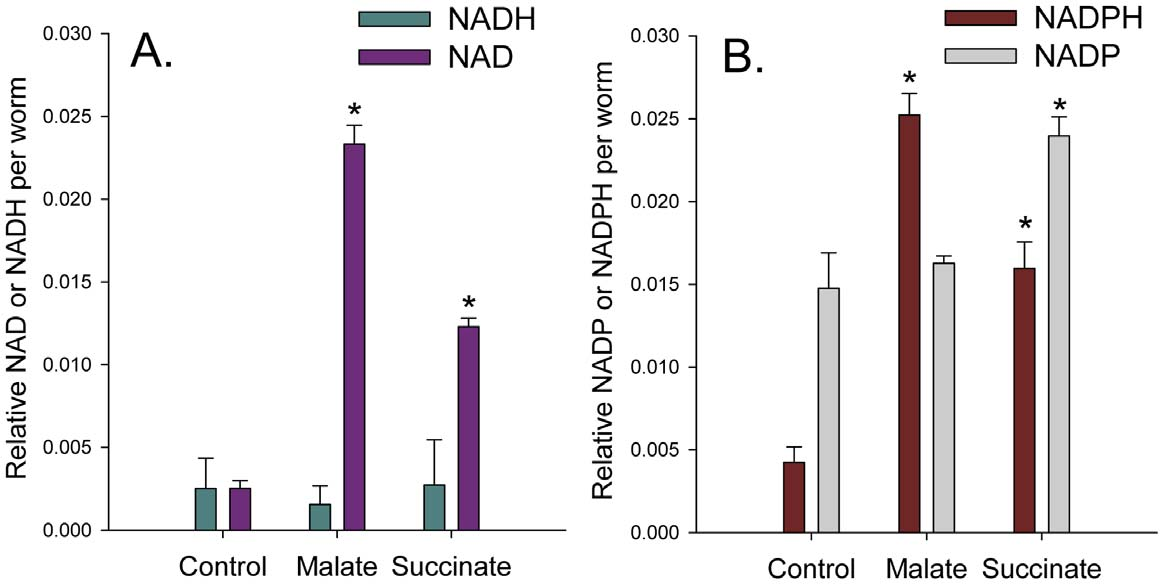
Malate treatment increased *C. elegans* NAD and NADPH levels more than succinate. A. Relative NAD and NADH levels in day 4 worms cultured with 10 mM malate, 10 mM succinate, or no addition (*p<0.05 compared to control). B. Relative NADP and NADPH levels in day 4 worms cultured with 10 mM malate, 10 mM succinate, or no addition (*p<0.05 compared to control).

An enhanced NAD/NADH ratio occurs in certain tissues during DR in rodents [8], and this ratio may be important for lifespan extension by activating sirtuins [40]. Therefore we cultured the worms for 4 days with malate or succinate and then measured NAD and NADH levels. As shown in Fig. 7A, malate addition greatly increased the NAD/NADH ratio. This result was surprising given that malate metabolism through the enzyme malate dehydrogenase converts NAD to NADH, which would yield opposite results. Malate addition also strongly increased total NAD+NADH levels, which also occurs in certain tissues during DR in mice [8]. Succinate, which did not extend lifespan, showed a smaller increase in NAD levels.

To determine if normal NAD(H) levels are required for malate-induced lifespan extension we individually knocked down two enzymes in the NAD synthesis pathway by RNAi and monitored lifespan (Table 1). We knocked down the NAD synthetase gene *qns-1* and the *nmnat-2* gene (W06B3.1) by RNAi. Knocking down W06B3.1 decreased lifespan by 6% and fully prevented lifespan extension by malate addition, suggesting normal NAD(H) levels may be necessary for malate-mediated lifespan extension. However, *qns-1* knockdown decreased lifespan by 24%, but malate addition increased lifespan by 13% (*p* = 0.07). This somewhat inconclusive data indicates further research is necessary to determine the exact role that NAD(H) levels play in malate-mediated lifespan extension.

We also measured NADP and NADPH levels following growth of *C. elegans* for 4 days with malate or succinate (Fig. 7B). Malate treatment greatly increased NADPH levels to decrease the NADP/NADPH ratio, while succinate treatment also increased NADPH levels, but to a lesser extent than malate. Both treatments also increased total NADP+NADPH levels.

### The Effects of Malate, Fumarate, and Succinate on Oxygen Consumption, ATP Levels, and Mitochondrial Membrane Potential

**Figure 8:**
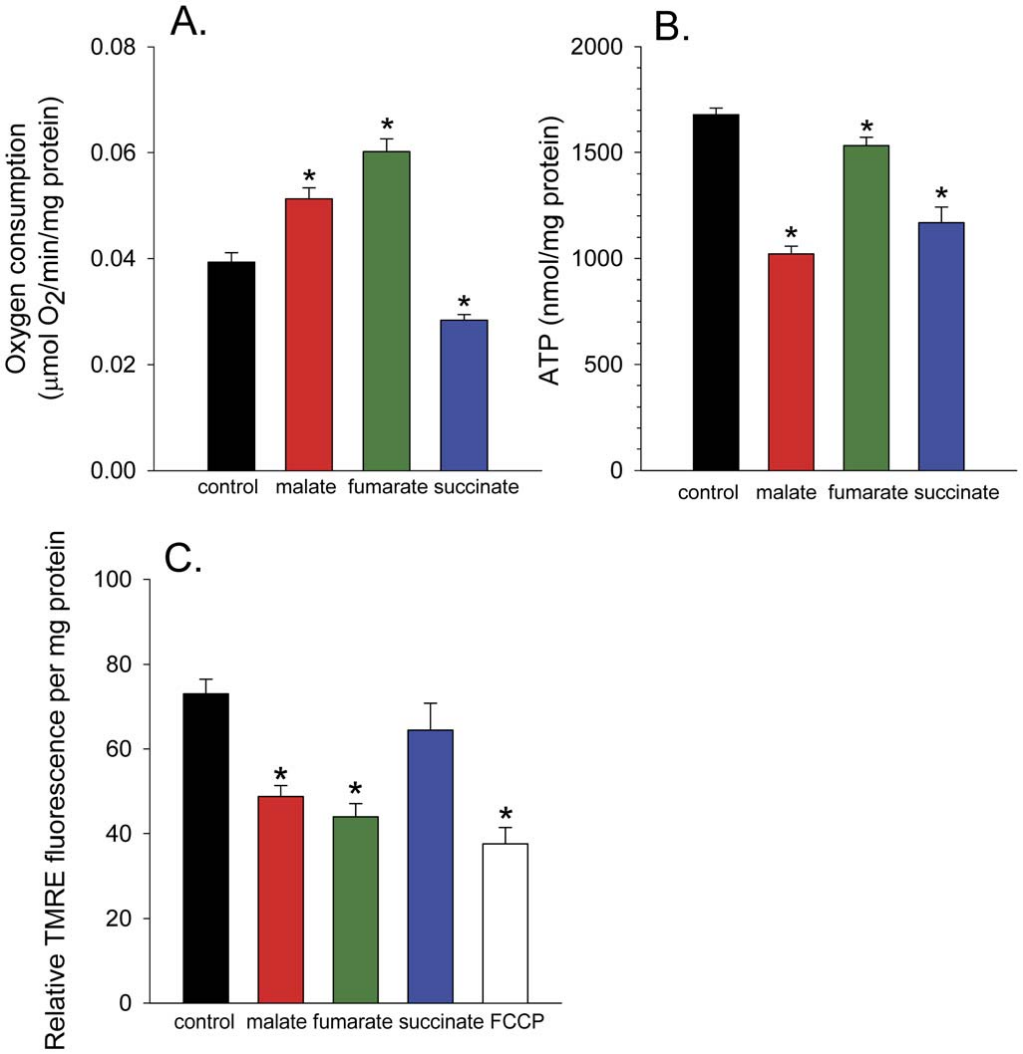
The effect of malate, fumarate, and succinate on respiration, ATP, and ΔΨ in *C. elegans*. (**A**) The effect of 10 mM malate, fumarate, or succinate treatment on oxygen consumption in day 4 N2 worms (*p*<0.001). (**B**) The effect of 10 mM malate, fumarate, or succinate treatment on ATP levels in day 4 N2 worms (*p*<0.001). (**C**) The effect of 10 mM malate, fumarate, or succinate or 10 µM FCCP treatment on ΔΨ in day 2 N2 worms.

To determine if malate, fumarate, or succinate treatment had an effect on mitochondrial function, we measured worm oxygen consumption (Fig. 8A), ATP levels (Fig.8B), and mitochondrial membrane potential (Fig.8C). Growth in the presence of malate for 4 days led to an increase in the rate of oxygen consumption. Growth in the presence of fumarate also increased respiration, while growth in the presence of succinate decreased respiration. Culture of the worms with malate or succinate greatly decreased ATP levels, while the decrease of ATP following culture with fumarate was small. To determine if TCA cycle metabolite-treated worms increased muscle contraction to burn more ATP, we conducted thrashing experiments. Malate treated worms showed a slightly decreased rate of thrashing (84.8±2.2 SEM body bends per minute) compared to controls (94.4±4.0 SEM body bends per minute) (*p* = 0.04) while fumarate and succinate treatment resulted in no significant difference in the rate of thrashing (87.6±3.3 and 92.8±3.4 SEM body bends per minute, respectively) compared to controls. Therefore, decreased ATP levels are not a result of increased thrashing.

One possible interpretation of the respiratory and ATP results is that malate and fumarate are inducing a mild uncoupling of oxidative phosphorylation. Therefore, we monitored the mitochondrial membrane potential (ΔΨ) with the cationic fluorescent dye TMRE (Fig. 8C). Worms grown in the presence of malate, fumarate, or the uncoupler FCCP showed decreased ΔΨ compared to control, with malate showing a robust decline, almost to the extent of FCCP. A non-toxic 10 µM concentration of uncoupler, a concentration that was previously shown to extend lifespan [41] was chosen. Succinate treatment showed a non-significant decrease in ΔΨ. Therefore, mitochondrial uncoupling is likely the cause for the decreased ΔΨ and ATP levels occurring following treatment with malate and fumarate, while decreased respiratory activity is likely responsible for the decreased ATP levels in succinate treated worms.

### Prevention of the Malate-Induced Drop in ATP Levels in *aak-2*, *sir-2.1*, and *hif-1* Mutants and in *gei-7* RNAi Knockdown Worms

To discern more about the malate-induced drop in ATP levels and to gain insight into possible important upstream players of malate-induced signaling pathways, we measured ATP levels of different mutant and RNAi knockdown worms grown in the presence or absence of malate (Table 2). We discovered that malate addition caused a large increase in the ATP levels in *aak-2(ok524)* and *sir-2.1(ok434)* mutants, while a small increase in ATP levels was observed in the *hif-1(ia4)* mutant and *gei-7* RNAi knockdown worms. Malate treatment decreased ATP to varying extents in the *daf-16(mgDf50)* and *hsf-1(sy441)* mutant strains and the *skn-1*, *sdha-2*, and *fum-1* RNAi knockdown worms. This data suggests that SIR-2.1 and AAK-2 may be important upstream transducers of malate signaling.

**Table 2:** Effect of 10 mM malate on *C. elegans* ATP levels.

Strain	RNAi knockdown	ATP following 10 mM malate (% of same strain untreated)	Standard error
N2 control		60.4%	3.3%
*daf-16(mgDf50)*		38.7%	1.8%
N2	*skn-1*	62.4%	1.9%
N2	*sdha-2*	64.4%	4.1%
*hsf-1(sy441)*		84.5%	3.0%
N2	*fum-1*	88.4%	2.7%
N2	*gei-7*	114.6%	2.4%
*hif-1(ia4)*		118.2%	2.5%
*sir-2.1(ok434)*		149.9%	2.8%
*aak-2(ok524)*		166.0%	5.1%

a Worms counted refers to the sum of the numbers counted on the first count day.

b α-ketoglu = α-ketoglutarate. *^c^*Performed in cell culture inserts.

ATP levels were measured as indicated in the Methods.

## Discussion

Mitochondrial electron transport chain function, which oxidizes NADH and FADH_2_, decreases with age across species [42]. This leads to a decreased cellular NAD/NADH ratio in specific tissues in aged organisms. Anti-aging therapies such as DR increase the NAD/NADH ratio in many tissues as a possible mechanism to delay aging. We show for the first time that malate and fumarate addition extend lifespan in *C. elegans*, while succinate addition did not. Addition of the TCA cycle intermediates increased the NAD/NADH ratio, which may be important for the mechanism of lifespan extension. Malate and fumarate treatment also increased oxygen consumption and decreased ΔΨ, suggesting a mild mitochondrial uncoupling, while succinate treatment did not. The glyoxylate shunt and malate dismutation/fumarate reduction metabolic pathways were also necessary for lifespan extension. Activation of these pathways together with induction of mitochondrial uncoupling likely result in increased cellular NAD levels. Increased NAD levels have been described to activate the histone deacetylase SIR-2.1 [30] and AMP kinase [43] to increase lifespan.

### Flavin and Pyridine Nucleotide Levels in Aging and Lifespan Extension

Since fumarate conversion to succinate by fumarate reductase also oxidizes FADH_2_ to FAD, an increased FAD/FADH_2_ ratio may play a role in lifespan extension. Malate and fumarate likely induce large increases in the FAD/FADH_2_ and NAD/NADH ratios to extend lifespan, while succinate has a smaller effect on the NAD/NADH ratio and likely an opposite effect on the FAD/FADH_2_ levels. In this regard, administration of 5 µM FAD to a short-lived *C. elegans* frataxin RNAi knockdown strain extended lifespan to an extent that surpassed the untreated control worms [44]. We also have obtained data that FAD addition to the medium extends lifespan (manuscript in preparation). FAD levels have been shown to decrease in many different tissues with age in rats [45], and levels were restored by exercise [46], which extends mean lifespan [47], [48].

Fumarate reductase has been shown to be essential for the growth of *Sacchromyces cerevisiae* under anaerobic conditions for the re-oxidation of FADH_2_ [49]_._ During the dauer state and other conditions that extend lifespan, *C. elegans* transitions to a metabolic state very similar to the one it enters during anaerobic conditions [50]. In dauer larvae, fumarate reductase activity and the glyoxylate cycle protein GEI-7 are upregulated [20], which decreases the amount of NAD reduced to NADH in the TCA cycle. However, oxygen is present under these conditions and electron transport chain complex I function continues to oxidize NADH. This metabolic transition increases the NAD/NADH ratio and may result in lifespan extension.

### Malate and Fumarate may Increase Lifespan through Increasing the NAD/NADH Ratio

Malate likely increases NADPH levels through the action of malic enzyme, converting malate to pyruvate with reduction of NADP to NADPH. Malate, as a TCA cycle intermediate, increases TCA cycle flux and electron transport chain activity to increase oxygen consumption. However, the results that malate increased the NAD/NADH ratio and decreased ATP levels were quite unexpected and may be key to the mechanism of lifespan extension induced by malate. Since oxygen consumption was increased and ΔΨ was decreased by malate and fumarate, they likely induce mitochondrial uncoupling. Uncoupling decreases ΔΨ, which often leads to reduced reactive oxygen species production. Mitochondrial uncouplers have been shown to extend lifespan in *C. elegans* [41], [51], consistent with the “uncoupling to survive” hypothesis of longevity [52].

Malate and fumarate may also increase lifespan by increasing mitochondrial respiration. Increased electron transport chain function relative to TCA cycle function will increase the NAD/NADH ratio, which may extend lifespan. In this regard, one research group has found a positive correlation between *C. elegans* oxygen consumption and lifespan. By examining lifespan following RNAi knockdown of the frataxin gene, the authors proposed that 73% of the lifespan decline following frataxin knockdown was due to decreases in the oxygen consumption rate [53]. This research group suggests that high rates of respiration are necessary to produce the normal reactive oxygen species-mediated cell signaling required for a normal lifespan. They further went on to show that glucose restriction increases lifespan by stimulating mitochondrial respiration [54] and that *daf-2* mutants show reduced glucose uptake, which stimulates mitochondrial oxidation of L-proline to increase oxygen consumption and increase lifespan [55]. Malate and fumarate could also extend lifespan by decreasing the rate of decline of oxygen consumption over the lifespan. In this regard, a research group using eight different long and short-lived mutant strains, found a strong correlation between the inverse of the rate of decline of oxygen consumption with age and the lifespan [56]. For example, long-lived *daf-2* worms showed a very slow rate of loss of oxygen consumption over their lifespan.

Another mechanism through which malate may increase the NAD/NADH ratio is through increasing the activity of the ETC, so more NADH is oxidized by complex I. This may be possible by activating the NADH-fumarate reductase (malate dismutation) system. Using this system, following oxidation of NADH by complex I, electrons can be passed to rhodoquinone instead of ubiquinone. Rhodoquinone passes electrons to membrane bound fumarate reductase (complex II), which terminally reduces fumarate to succinate. In order for this activity to lead to oxidation of NADH at a faster rate, complex I activity must be limited by the amount of oxidized coenzyme Q (ubiquinone). If this is true, increasing the amount of oxidized rhodoquinone by increasing fumarate levels could increase complex I activity to increase the NAD/NADH ratio. Using fumarate as a terminal electron acceptor would also result in decreased ATP levels as only one proton is pumped per NADH oxidized instead of 3 protons being pumped when oxygen is used as the terminal electron acceptor. Decreased electron flow through complex III of the ETC could decrease ROS production and be a mechanism of lifespan extension, as complex III is an important generator of ROS [57]. However, since malate addition increased oxygen consumption in the worms, fumarate reduction likely only plays a minor role in total ETC function under these conditions.

Glyoxylate shunt activity also increases the NAD/NADH ratio as the shunt bypasses two of the three NADH generating reactions of the TCA cycle. We have also shown that glyoxylate shunt activity is required for the malate or fumarate-mediated increase in lifespan. The glyoxylate shunt gene *gei-7* has been shown to be required for lifespan extension mediated by *daf-16* in *daf-2* insulin receptor mutants [58]. So it is not surprising that the glyoxylate shunt is also required for the lifespan extension mediated by TCA cycle metabolites, which is also *daf-16* dependent.

### Does Malate Increase Acetyl-CoA Levels to Increase Lifespan?

The glyoxylate shunt conversion of malate and CoA to glyoxylate and acetyl-CoA may be important for malate-mediated lifespan extension. Other metabolites that potentially increase acetyl-CoA levels, such as pyruvate [15] and acetate [14], have also been shown to increase lifespan. Further support of an important role of increased acetyl-CoA levels in lifespan extension is that glyoxylate addition did not extend lifespan (Figure S3). Glyoxylate can be converted to malate, but at the expense of decreasing acetyl-CoA levels. DR induces a metabolic shift from glucose oxidation to fatty acid oxidation that would also increase acetyl-CoA levels. Histone acetyltransferases (HATs) utilize acetyl-CoA as a cofactor for acetylation of histone tails. In this regard the HAT *cpb-1*/p300 is induced in *daf-2* worms and by DR and is essential for full lifespan extension by these interventions in *C. elegans* [59]. The histone deacetylase inhibitors sodium butyrate and trichostatin A also increased lifespan in *C. elegans*. In yeast it has been demonstrated that acetyl-CoA levels regulate protein acetylation [60] and that prevention of the acetylation of the gluconeogenic enzyme PEPCK blocks chronological lifespan extension induced by water starvation [61].

### A Proposed Mechanism of How Malate Metabolism Results in Increased Lifespan

We hypothesize that addition of malate or fumarate to *C. elegans* somehow leads to activation of the glyoxylate shunt. Regulation of the glyoxylate shunt has not been well studied in eukaryotes. In Gram-negative bacteria, a dual function kinase/phosphatase AceK responds to changes in carbon source to control phosphorylation-induced inactivation of isocitrate dehydrogenase, which induces flux into the glyoxylate shunt [62]. Lysine acetylation of isocitrate lyase and AceK also regulate shunt activity [63]. Upregulation of shunt activity would increase NAD levels, which are known to activate AMP kinase [43] and sirtuins [64]. AMP kinase activation can further increase NAD levels and sirtuin activity [65]. However, metabolism under these conditions likely becomes limited by FAD levels, so malate dismutation is activated to oxidize FADH_2_ to FAD. SIR-2.1 is known to activate DAF-16 activity [66], which can lead to lifespan extension [67] and further upregulation of *gei-7* expression [58] to amplify the lifespan extending signaling pathway.

### Does Mitochondrial Uncoupling Play a Role in Malate-Mediated Lifespan Extension?

Malate-mediated mitochondrial uncoupling may be essential for lifespan extension. But 3 experimental results are inconsistent with this suggestion. First, malate addition resulted in lifespan extension in *hif-1* mutant worms, where ATP levels remain high, suggesting uncoupling is not occurring to a great extent in this strain, yet lifespan was still extended. Second, malate addition to *daf-16* mutants resulted in a large decrease in ATP levels, which may indicate mitochondrial uncoupling was occurring, when no lifespan extension was induced. However, one must be careful in ascribing decreases in ATP levels to decreases in oxidative phosphorylation. Changes in glycolysis and buffering ATP into phosphocreatine can also cause relatively quick changes in ATP levels without altering oxidative phosphorylation. And third, lifespan extension mediated by the uncoupler CCCP was described to be *daf-16* independent [41], whereas the lifespan extension mediated by malate requires *daf-16*. Further research needs to be performed to determine if activation of mitochondrial uncoupling, or, minimally, a decreased ΔΨ, is a common pathway of lifespan extension for compounds that extend *C. elegans* lifespan. In this regard we have found that a blueberry/green tea extract mixture that extended *C. elegans* lifespan also increased oxygen consumption and decreased ATP levels (data not shown).

### Malate-Induced Lifespan Extension Compared to Oxaloacetate-Induced Lifespan Extension

Unsurprisingly, the lifespan extension observed following malate addition was similar to that observed with oxaloacetate treatment [13]. For example, both required *daf-16*. However, there were slight differences. Oxaloacetate was reported to extend median lifespan by 25%, while we report malate only increased mean lifespan by 14%. Under our liquid culture conditions we found that 10 mM oxaloacetate extended mean lifespan by 49% (data not shown). This larger effect than malate or fumarate may be due to a higher NAD/NADH ratio in oxaloacetate fed worms. Also, we found that malate-induced lifespan extension was completely dependent upon the presence of *sir-2.1*, while oxaloacetate-induced lifespan was still increased in the absence of *sir-2.1* [13]. This may be due to different growth conditions, either in liquid or on agar medium.

The worms in most of our experiments were cultured in liquid S medium, which differs slightly in nutrient composition from nematode growth media (NGM) commonly used for culturing worms on agar. The liquid S medium contains 10 mM citrate (a TCA cycle metabolite), in addition to phosphate as a buffer, while the NGM agar lacks citrate, but contains peptone powder (2.5 g/l) absent in S medium. The added citrate may not be required for malate-mediated lifespan extension as we found that malate extended the lifespan of worms grown on NGM agar plates by 10% (see Table 1), but this should be further verified due to the small number of worms used in the experiment. Also, it has been reported that adding citrate to the culture medium did not extend lifespan [14]. The worms grown in liquid medium are not dietarily restricted as *eat-2* worms showed a robust increase in lifespan in liquid medium, as they do on agar plates.

### TCA Cycle Function is a Key Determinant of Longevity

Much data implicate TCA cycle function in the control of longevity. Many TCA cycle genes are upregulated in long-lived Ames dwarf and Little mice [68]. Brown Norway rats, a long-lived strain, do not shown declines in brain TCA cycle function with age in contrast to short-lived strains [69]. In yeast, glucose limitation increases chronological lifespan and upregulates TCA cycle gene expression [70]. Yeast mitochondrial ETC gene knockouts do not show extended chronological lifespan under DR conditions, but most TCA cycle gene knockouts showed even greater extension of lifespan than the wild-type yeast undergoing DR [71]. Yeast mutants with increased lifespan had increased levels of TCA cycle metabolites [72]. A downregulation of TCA cycle and ETC gene expression occurs in long-lived *C. elegans* dauer larvae [19] while long-lived *daf-2* insulin receptor mutants show either unchanged [19] or decreased [73] TCA cycle gene expression with either unchanged [19] or increased [73] ETC gene expression. Mutations in the *Drosophila* Indy gene [74], a sodium coupled TCA cycle dicarboxylate and tricarboxylate carrier in the plasma membrane extend lifespan. Similar results were found when two of the three *C. elegans* homologs of Indy were knocked down [75], [76]. But others failed to replicate these findings [77]. Knockdown of the mouse homolog of Indy resulted in DR-like phenotypes as well [78]. As a whole, there appears to be little consistency in these observations in different experimental models. However, proper coordination between ETC function and TCA function is likely necessary to maintain a normal to slightly high NAD/NADH ratio conducive to long life. High TCA cycle function with low ETC function is not favorable for extended lifespan because this would drive down mitochondrial and cellular NAD/NADH slowing important NAD-driven reactions likely necessary for extended lifespan. As another example, dietary restriction in mammals likely decreases flux through the TCA cycle while ETC function is maintained throughout lifespan, resulting in an increased NAD/NADH ratio in several important tissues and lifespan extension.

### Succinate as a Blocker of DR-Induced Longevity

Since succinate, but not malate addition blocked lifespan extension in *eat-2* worms, it is possible that reduction of fumarate to succinate or maintaining a high FAD/FADH_2_ ratio is essential for DR-induced longevity in *C. elegans*. Increased succinate levels likely decrease fumarate reductase activity through product inhibition. However, since long-lived *eat-2* worms were shown to have a 21-fold increase in the rate of [2-^14^C] acetate oxidation as measured by ^14^CO_2_ release [79], perhaps *eat-2* worms increase both TCA cycle activity (at least the CO_2_ generating portion of the cycle) and fumarate reduction to extend lifespan. Interestingly, proteomic experiments revealed that the glyoxylate cycle protein GEI-7 was down-regulated slightly in *eat-2* worms [79], while GEI-7 is upregulated in long-lived dauer and *daf-2* worms [19]. Therefore distinct metabolic programs may be activated to extend lifespan under these different conditions.

### Malate Treatment Has Beneficial Effects in Mammals

Although we showed an important role for the glyoxylate shunt and malate dismutation, metabolic pathways absent in mammals, in malate-mediated lifespan extension in *C. elegans*, malate treatment has been shown to be very beneficial in mammals as well. Malate is found at high concentrations in unripened fruit, most notably in apples, and may contribute to some of the beneficial effects when these fruit are consumed. In addition, livers excised from aged rats that had been administered malate for 30 days displayed increased activities of complexes I, III, and IV of the electron transport chain (ETC) [80]. Malate also improved antioxidant function, leading to increased superoxide dismutase, glutathione peroxidase, reduced glutathione, and decreased lipid peroxidation [81]. Malate supplementation also increased the activity of malate-aspartate shuttle components [82]. However, malate had no effect on the decreased mitochondrial membrane potential measured in aged rat liver [80]. Therefore, many of the protective effects of malate treatment seem to be conserved from nematodes to mammals.

### Conclusion

Malate and fumarate increased the lifespan of *C. elegans*, while succinate did not. The glyoxylate shunt and malate dismutation/fumarate reduction pathways and SIR-2.1 were required for malate-mediated lifespan extension. DAF-16 translocation to the nucleus and transcription of DAF-16 target genes also plays an essential role in malate-mediated lifespan extension. However, since succinate addition can also induce DAF-16 nuclear translocation without lifespan extension, other factors are also involved. In this regard, further research should aim to elucidate the mechanisms through which addition of malate and fumarate to the culture medium lead to an uncoupling of mitochondrial oxidative phosphorylation and also determine if the FAD/FADH_2_ ratio plays a role in lifespan determination. Since fumarate, malate, and oxaloacetate extend lifespan in *C. elegans* through a mechanism similar to dietary restriction, an anaplerotic cocktail of these compounds may be useful for the treatment of human aging-associated disorders.

## Supporting Information

Figure S1 **α-ketoglutarate addition did not alter the lifespan of** ***C. elegans*** **.** *C. elegans* N2 worms were grown in cell culture inserts in 12-well microplates fed heat-killed *E. coli* with media change every 3 days in the absence or presence of 10 mM α-ketoglutarate (log-rank *p* = 0.21 vs. untreated control).
(TIF)

Figure S2 **Aspartate addition did not alter the lifespan of** ***C. elegans*** **.** *C. elegans* N2 worms were grown in cell culture inserts in 12-well microplates fed heat-killed *E. coli* with media change every 3 days in the absence or presence of 10 mM aspartate (log-rank *p* = 0.57 vs. untreated control).
(TIF)

Figure S3 **Malate and fumarate increased the lifespan of** ***hif-1*** **mutant worms.** *C. elegans hif-1(ia4)* worms were grown in cell culture inserts in 12-well microplates fed heat-killed *E. coli* with media change every 3 days in the absence or presence of 10 mM malate (log-rank *p*<0.001 vs. untreated control) or 10 mM fumarate (log-rank *p* = 0.02 vs. untreated control).
(TIF)

Figure S4 **Glyoxylate addition did not alter the lifespan of** ***C. elegans*** **.** *C. elegans* N2 worms were grown in cell culture inserts in 12-well microplates fed heat-killed *E. coli* with media change every 3 days in the absence or presence of 10 mM glyoxylate (log-rank *p* = 0.35 vs. untreated control).
(TIF)
