# Supplementary figures and images for: Malate and Fumarate Extend Lifespan in Caenorhabditis elegans
Source: PLoS One. 2013 Mar 5;8(3):e58345. doi: 10.1371/journal.pone.0058345 (PMC3589421; doi:10.1371/journal.pone.0058345)

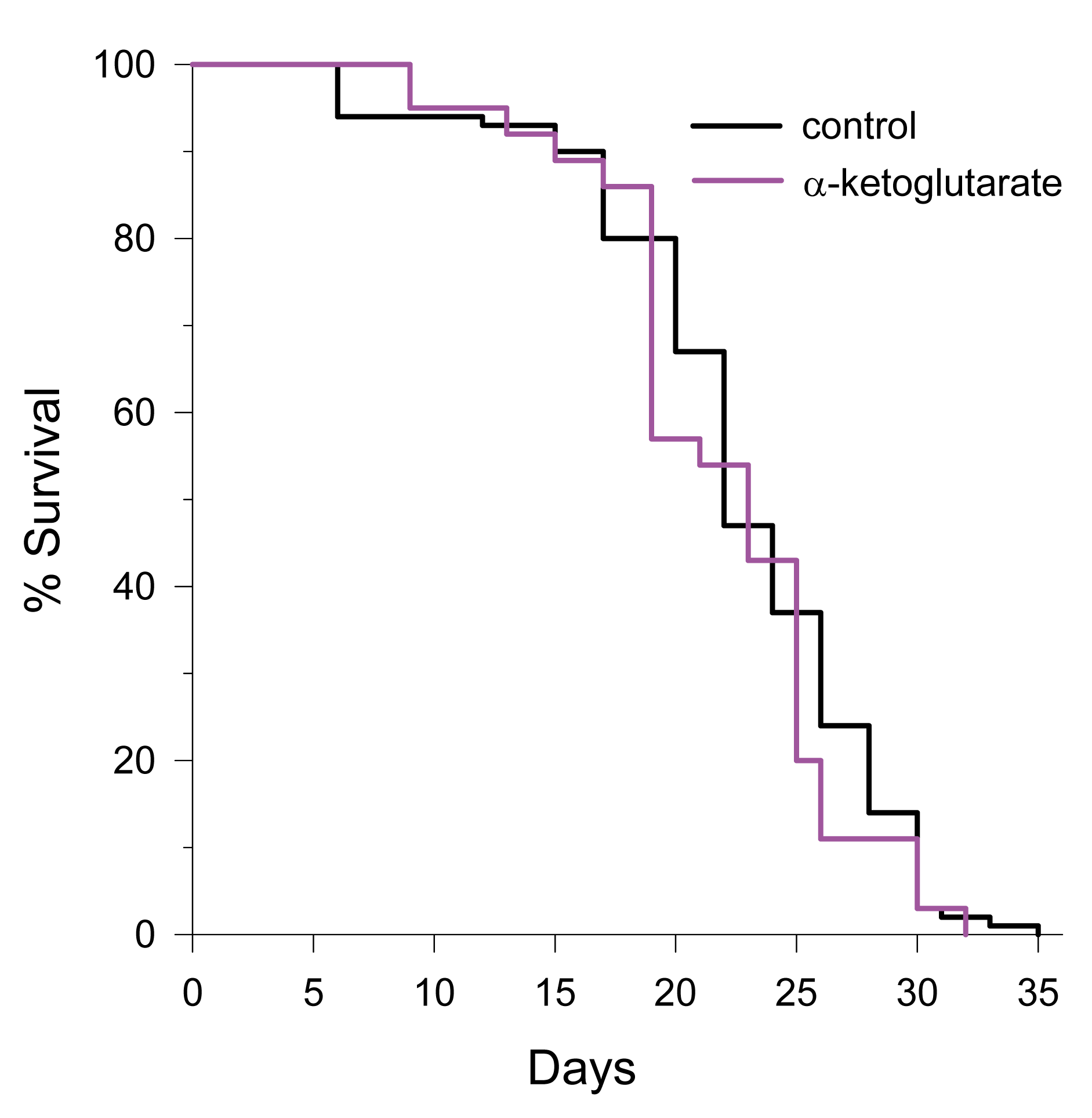

Supplement: Figure S1 — α-ketoglutarate addition did not alter the lifespan of C. elegans . C. elegans N2 worms were grown in cell culture inserts in 12-well microplates fed heat-killed E. coli with media change every 3 days in the absence or presence of 10 mM α-ketoglutarate (log-rank p = 0.21 vs. untreated control). (TIF) [file pone.0058345.s001.tif]

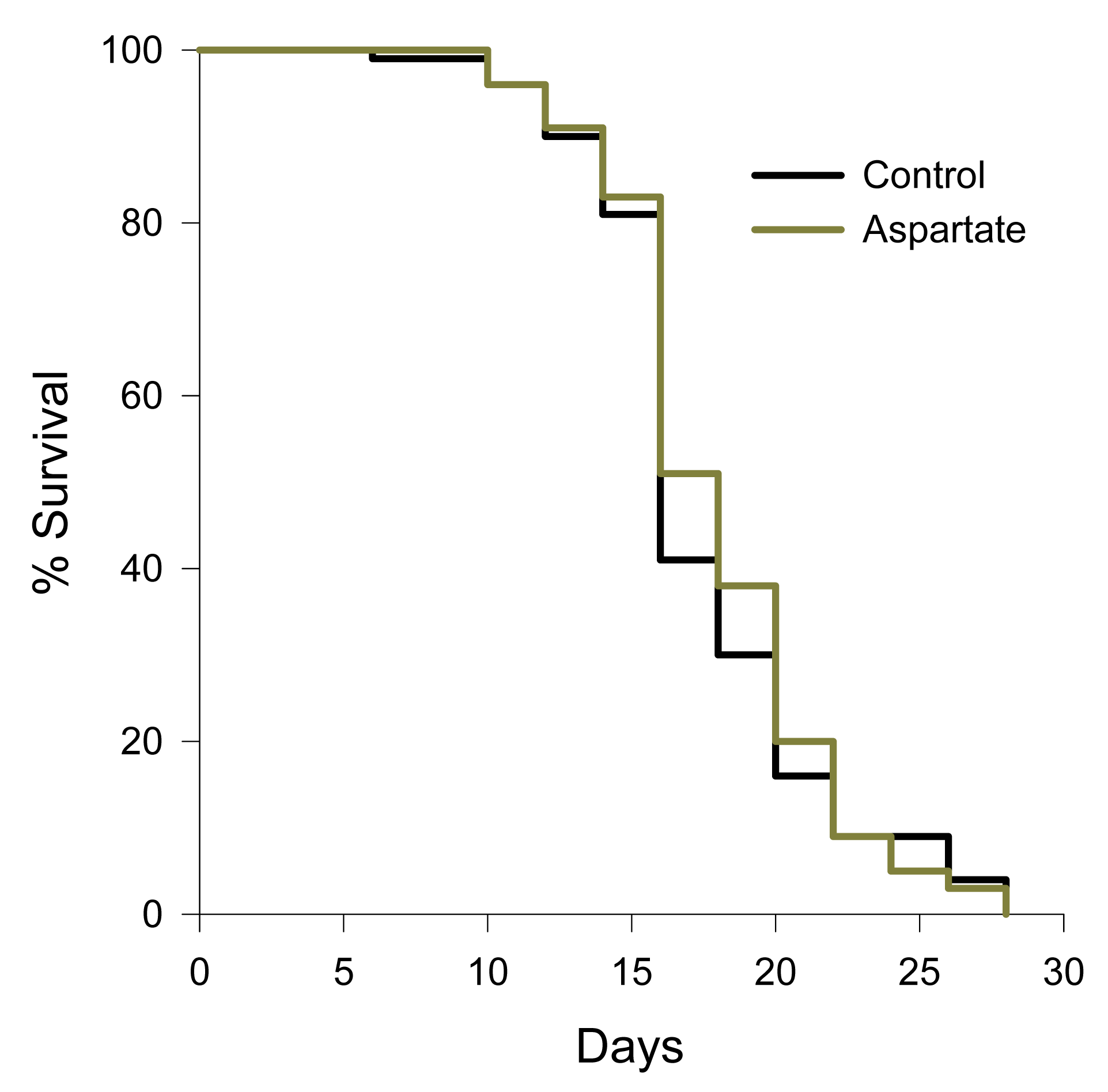

Supplement: Figure S2 — Aspartate addition did not alter the lifespan of C. elegans . C. elegans N2 worms were grown in cell culture inserts in 12-well microplates fed heat-killed E. coli with media change every 3 days in the absence or presence of 10 mM aspartate (log-rank p = 0.57 vs. untreated control). (TIF) [file pone.0058345.s002.tif]

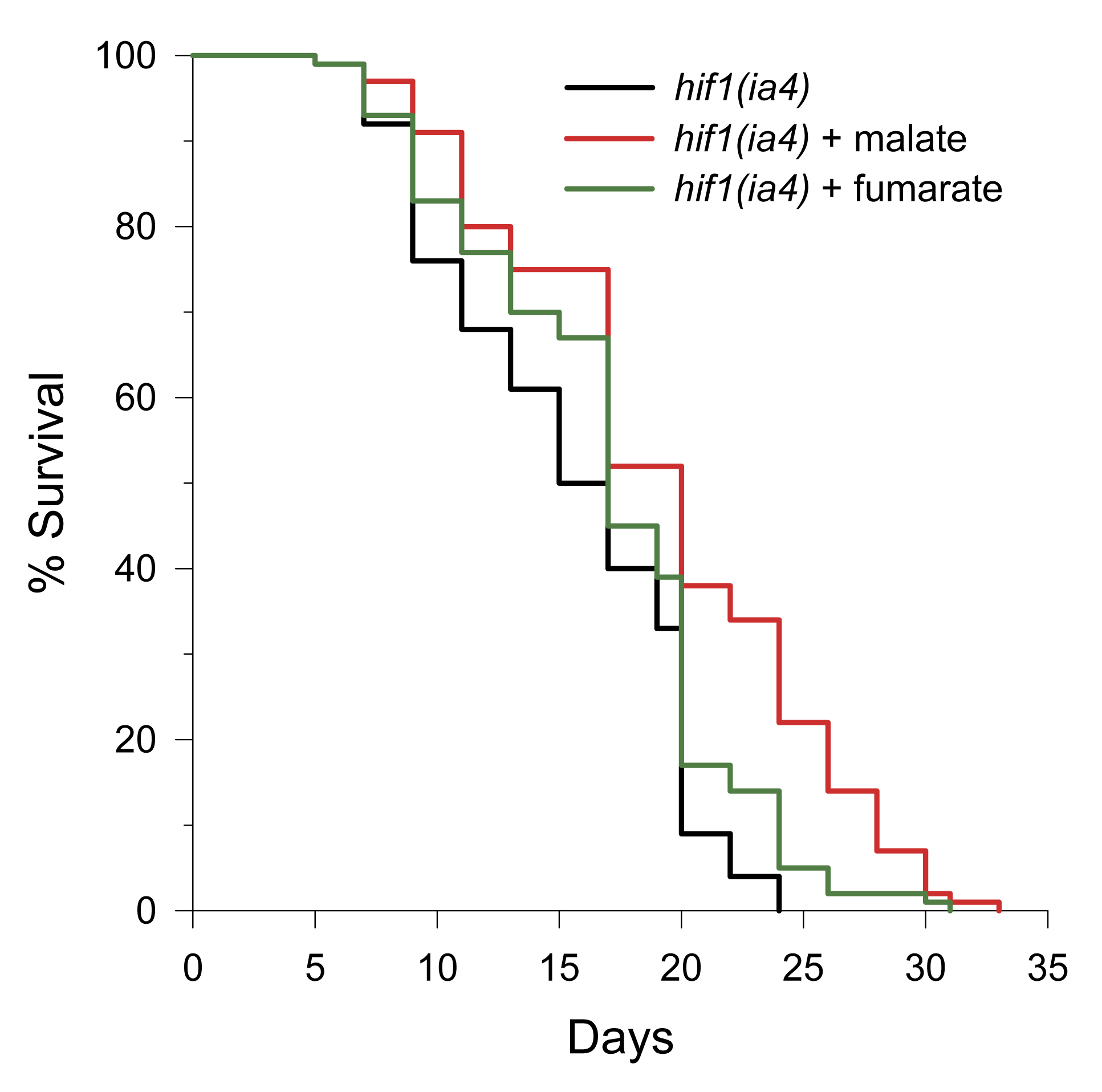

Supplement: Figure S3 — Malate and fumarate increased the lifespan of hif-1 mutant worms. C. elegans hif-1(ia4) worms were grown in cell culture inserts in 12-well microplates fed heat-killed E. coli with media change every 3 days in the absence or presence of 10 mM malate (log-rank p<0.001 vs. untreated control) or 10 mM fumarate (log-rank p = 0.02 vs. untreated control). (TIF) [file pone.0058345.s003.tif]

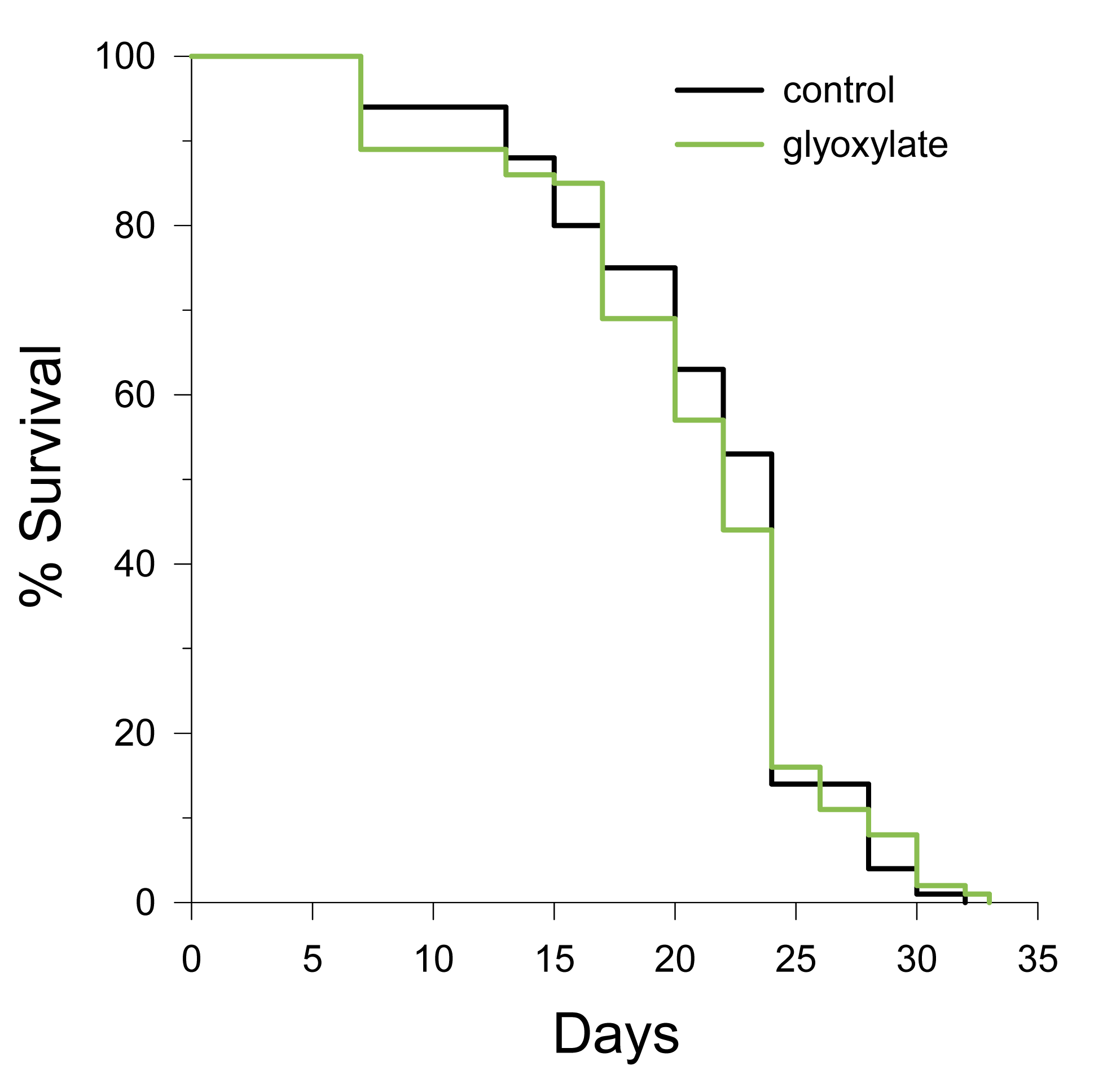

Supplement: Figure S4 — Glyoxylate addition did not alter the lifespan of C. elegans . C. elegans N2 worms were grown in cell culture inserts in 12-well microplates fed heat-killed E. coli with media change every 3 days in the absence or presence of 10 mM glyoxylate (log-rank p = 0.35 vs. untreated control). (TIF) [file pone.0058345.s004.tif]
